# Supplementary material for: Arabidopsis FORGETTER1 mediates stress-induced chromatin memory through nucleosome remodeling
Source: eLife. 2016 Sep 28;5:e17061. doi: 10.7554/eLife.17061 (PMC5040591; doi:10.7554/eLife.17061)
Supplement: Supplementary file 1. — DOI: http://dx.doi.org/10.7554/eLife.17061.027 [file elife-17061-supp1.docx]

**Supplemental Table S1: Sequences of oligonucleotides used in this study.**

| Primer name | Sequence |
| --- | --- |
| MNase |  |
| 1913/HSP22_TSS-393_F | GACACAAGCATGGCAAGCCAA |
| 1914/HSP22_TSS-393_R | TGACCTCTATTGCCCTATG |
| 1901/HSA32_TSS-216_F | ACCTTTTCAATATTCGATT |
| 1902/HSA32_TSS-216_R | CAGCGATTCAAAACGCAAAC |
| 1903/HSA32_TSS-175_F | GAAGAGGACGTTTGCGTT |
| 1904/HSA32_TSS-175_R | CTGTAAAGGCCCACTTAAAT |
| 1905/HSA32_TSS-124_F | CCAGTCGCTCTGGCCCAAT |
| 1906/HSA32_TSS-124_R | GTTCTCGAATTTGTTACTCT |
| 1907/HSA32_TSS-75_F | TATCCTAAATCCCGTCTCGTT |
| 1908/HSA32_TSS-75_R | ATGTCCAAGTCGAAGAATGG |
| 1893/HSA32_TSS+57_F | TTCGAGAAATGTTCGATTCT |
| 1894/HSA32_TSS+57_R | CCATCTGTAGTAAGCCGCCA |
| 1895/HSA32_TSS_F | ATATTTCTCTAGAAGCAGCT |
| 1896/HSA32_TSS_R | TCGAAGAATCGAACATTTCT |
| 1909/HSA32_TSS+133_F | AGAAGTGAACAGAGAAAATG |
| 1910/HSA32_TSS+133_R | CATCTCCGTAACACCGTAAC |
| 1911/HSA32_TSS+194_F | GTTACGGTGTTACGGAGATG |
| 1912/HSA32_TSS+194_R | TGTGAATCATAACCGGAATA |
| 1913/HSP22_TSS-393_F | GACACAAGCATGGCAAGCCAA |
| 1914/HSP22_TSS-393_R | TGACCTCTATTGCCCTATG |
| 1919/HSP22_TSS-130_F | AGGCCCAATCCAATCCATAG |
| 1920/HSP22_TSS-130_R | GGCTGTGACTTGTACTAATT |
| 1921/HSP22_TSS-60_F | CACAACAAACTTATCCAACG |
| 1922/HSP22_TSS-60_R | GGTAGAGTTTTGCAGAGAGA |
| 1923/HSP22_TSS-8_F | GCTAGAACAATCTCAATATC |
| 1924/HSP22_TSS-8_R | GATGGTTAGTCTCAATTCTC |
| 1925/HSP22_TSS+93_F | GAGAATTGAGACTAACCATC |
| 1926/HSP22_TSS+93_R | TGTAGAAGGTTGAAACCAA |
| 1927/HSP22_TSS+155_F | TTGGTTTCAACCTTCTACA |
| 1928/HSP22_TSS+155_R | GACCCTTCACTTGTTTTGATG |
| 1930/HSP22_TSS_+327_F | ATCCGATCTCTGGCTAGACC |
| 1931/HSP22_TSS_+327_R | AGACAGAGCCACGCTTGTGT |
| 1932/HSP22_TSS_+401_F | CACAAGCGTGGCTCTGTCT |
| 1933/HSP22_TSS_+401_R | ACTTCATCCTTCTTCAAACC |
| 1934/HSP22_TSS_+437_F | ATGAGATAATGCTCGATATC |
| 1935/HSP22_TSS_+437_R | CTCCACTGACTCGTAAAACT |
| 1936/HSP18.2_TSS_-367_F | TTGTCCTCTTTTCTCTGAG |
| 1937/HSP18.2_TSS_-367_R | TCGAGATACGGGCTCAGTGA |
| 1938/HSP18.2_TSS_-262_F | AGGATAATACAACAACAAAG |
| 1939/HSP18.2_TSS_-262_R | CACTGTGGTGAAATGACCAG |
| 1940/HSP18.2_TSS_-125_F | TATGTGTTCTAAAGACTCCA |
| 1941/HSP18.2_TSS_-125_R | GTTAGAGGATGAAGAGAGAA |
| 1942/HSP18.2_TSS_-48_F | TTCTCTCTTCATCCTCTAAC |
| 1943/HSP18.2_TSS_-48_R | CGTTGCTTTTCGGGAGACTT |
| 1944/HSP18.2_TSS_+32_F | AAGTCTCCCGAAAAGCAACG |
| 1945/HSP18.2_TSS_+32_R | ATCCCATAAGTCTTGCGAGA |
| 1946/HSP18.2_TSS_+152_F | CATCTTCTGCGTTGGCAAAC |
| 1947/HSP18.2_TSS_+152_R | CCGGCGTTTCCTTCCAATCC |
| 1948/HSP18.2_TSS_+216_F | GGATTGGAAGGAAACGCCGG |
| 1949/HSP18.2_TSS_+216_R | TTGCTCCTCTCTCCGCTAATC |
| 1950/HSP18.2_TSS_+410_F | GGTTACCGGAGAATGCAAAG |
| 1951/HSP18.2_TSS_+410_R | GGAGATATCGATGGACTTGA |
| 1806/At4g07700_1_F | ACTGGTTGCTAGCTGGGAGA |
| 1807/At4g07700_1_R | CCAGTGTTGGTTCTCCTTGG |
| 1808/At4g07700_53_F | TGGAGAAAGTGTTCTCGGATAAA |
| 1809/At4g07700_53_R | TTGATCTGAATAGTTGCATTGACA |
| 1810/At4g07700_101_F | GCGAAGTTGCTGTGAACAAA |
| 1811/At4g07700_101_R | TACATTGAGTTTGGCCGATG |
| 1812/At4g07700_186_F | CGGCCAAACTCAATGTAAGC |
| 1813/At4g07700_186_R | TCCCTCTTCTAGAGGTTTTGTCC |
| 2669/HSP101_TSS -348_F | TCCATCTGACTCCTTTAATT |
| 2670/HSP101_TSS -348_R | ATCAAAATCCAAAGATTTACG |
| 2671/HSP101_TSS -136_F | ACATCTACCTGTCGGATCAA |
| 2672/HSP101_TSS -136_R | TCTGGAAAGATAGAGAACTA |
| 2673/HSP101_TSS -33_F | TAGTTCTCTATCTTTCCAGA |
| 2674/HSP101_TSS -33_R | TCAAGCAGATTATAGCGGTA |
| 2675/HSP101_TSS _112_F | TCTGCTTGATTCTCTGCAA |
| 2676/HSP101_TSS _112_R | ACACACAAATGAGAACAAGA |
| 2677/HSP101_TSS _237_F | TCTTGTTCTCATTTGTGTGT |
| 2678/HSP101_TSS _237_R | GGATTCATCTTCGATTAGCT |
| 2679/HSP101_TSS _336_F | AGCTAATCGAAGATGAATCC |
| 2680/HSP101_TSS _336_R | ATCAAAGCACCAGCTAAATG |
| 2681/HSP101_TSS _444_F | CATTTAGCTGGTGCTTTGAT |
| 2682/HSP101_TSS _444_R | AGGTGGAGGAGATTGTGAAG |
| 2683/HSP101_TSS _568_F | CTTCACAATCTCCTCCACCT |
| 2684/HSP101_TSS _568_R | GACCCATAATCAACTGGTCA |
| ChIP |  |
| 800/4g21320R_genebody2 | AACACCGTTCAGCCTTTCTG |
| 801/4g21320F_genebody2 | CTCGGTCAAGCGGTAAGAAG |
| 1259/IG5_HSP22-3kb_FWD | CGTTGGACTTGGCCTTAGAT |
| 1260/IG5_HSP22-3kb_REV | TGACTGCTCCCTGATTCTTG |
| 2051/HSP18.2_TSS_-1068_F | CTTTAGTCAGGGCAGCTACA |
| 2052/HSP18.2_TSS_-1068_R | CATCACCAGCAAAATTCACTC |
| 796/act7_for | CGTTTCGCTTTCCTTAGTGTTAGCT |
| 797/act7_rev | AGCGAACGGATCTAGAGACTCACCTTG |
| 2225/Mu1C-ChIP-spe1f | TTCTTCTCCATCGACACTCTCTC |
| 2062/MU1C-A-rev | ATGATGATCCAACGACCAACCAC |
| Cloning |  |
| 1774/EMB1135_pENTY_F | ATGGTCGACTGGATCCAAATGACGCAGTCGCCTGTTCAAC |
| 1775/EMB1135_pENTY_R | AGCTGGGTCTAGATATCCTTAATCATCAATCTCTTGAA |
| 1778/CHR17_pENTRY_F | ATGGTCGACTGGATCCAAATGGCTAGAGCTTCGAAG |
| 1779/CHR17_pENTRY_R | CTGGGTCTAGATATCCTTATTTCCTACGCTTTCCCGAGCT |
| 1780/CHR11_pENTRY_F | ATGGTCGACTGGATCCAAATGGCGAGAAATTCGAAT |
| 1781/CHR11_pENTRY_R | AGCTGGGTCTAGATATCCTCATCTCATCGACAGGTGCT |
| 1859/BRM_pENTRY_F | ATGGTCGACTGGATCCAAATGCAATCTGGAGGCAGTGG |
| 1860/BRM_pENTRY_R | AGCTGGGTCTAGATATCCCGCTAAATGGCTAGGCCGTCT |
| 998/EMB1135PHD2_ERIF | GGATCCCCAGATAGTGATAATGAAAGTGA |
| 999/EMB1135PHD1_StpSalR | GTCGACTACCTTTTCTGCAATTCGGCAAT |
| 1166/AtING1_F_EcoRI | GAATTCTCAAGTAATATGGATCTGGA |
| 1167/AtING1_R_SalI | GTCGACTCATCGACCTTTCCT |
| qRT PCR |  |
| 1547/F-AT4G26410 | GAGCTGAAGTGGCTTCAATGAC |
| 1548/R-AT4G26410 | GGTCCGACATACCCATGATCC |
| 363/AT4G27670_qPCR_F | TGGACGTCTCTCCTTTCGGATTGT |
| 364/AT4G27670_qPCR_R | TGCACGAATCTCTGACACTCCACT |
| 359/AT5G59720_qPCR_F | ACAAACGCAAGAGTGGATTGGA |
| 360/AT5G59720_qPCR_R | GCTCCTCTCTCCGCTAATCTGC |
| 267/HSP101F | ATGACCCGGTGTATGGTGCTAG |
| 268/HSP101R | CGCCTGCATCTATGTAAACAGTG |
| 253/Hsa32F | CGGTCAAGCGAGTTTGTGGAGGA |
| 254/Hsa32R | GGAATCCGCGTATTTGCAGACATCA |
| 354/LP-LUC | GCAAGGATATGGGCTCACTG |
| 647/LUC-R | CACACACAGTTCGCCTCTTTG |
| 2302/Hsp21_unsp _F | TTAATCTAACCACAGGATTGTTGGATC |
| 265/HSP70F | CCGTCTTCGATGCTAAGCGTCT |
| 266/HSP70R | AACCACAATCATAGGCTTCTCACC |
| 316/HSP70uspl_F | GGAGGAAGTAAGTATTAAACTTGTTGTT |
| 2725/HSP101_unsp_F | CTGACTCTTGTGGTTGCTTTCT |
| 637/AT4G10250 REV | TTCAGGAGATAGTTTCGTGAGGTTA |
| 638/AT4G10250 FOR | ATTCTGGAGACAGTTCAAGCTACCT |
| 315/Hsa32uspl_F | TATGCTTACTGTGAGAATGCCTTTGT |
| 653/HSA32-3UTR | TTCTTACAGCATCAAAGAAGCA |
| 358/HSA32_qPCR-F | TGGTCCAAATGTGAATCTCTATGT |
| 939/EMB1135-qPCR-F | TGTCTGGTGCCTCAACTATGCTC |
| 940/EMB1135-qPCR-R | CTAGCATTGAACTCGCAGACTCC |
| Hbo84/At1g13320-qPCRfor | GCATTTCACTCCTCTGGCTAAG |
| Hbo84/At1g13320-qPCRrev | GGCACTTGGGTATGCAATATG |
| 844/EMB1135BamHI_F | AGGGATCCACAATGACGCAGTCGCCTGTTCAAC |
| 845/EMB1135BamHI_R | TATGGATCCTTAATCATCAATCTCTTGAACCCATGC |
| Genotyping |  |
| 1110/FLAG_268E09_LP | CATCAAATTCTGGTCCACACC |
| 1111/FLAG_268E09_RP | TTGCCAGGTTGCTTTTAAATG |
| 1420/LBb1 | GCGTGGACCGCTTGCTGCAACT |
| 1117/SALK_036520_LP | TTAAGCGCTGGAAAACGTATC |
| 1892/SALK_036520_RP | AAGCACAAGGATGAAAAGCAG |
| 1886/SALK_Lba | TGGTTCACGTAGTGGGCCATCG |
